# Editorial: Self-assembly of biomolecules as healthcare materials: drug delivery and beyond

**DOI:** 10.3389/fchem.2024.1473033

**Published:** 2024-08-19

**Authors:** Weiqi Zhang, Benedict Law

**Affiliations:** ^1^ State Key Laboratory of Complex Severe and Rare Diseases, Institute of Basic Medical Sciences, Chinese Academy of Medical Sciences and Peking Union Medical College, Beijing, China; ^2^ Molecular Imaging Innovations Institute, Department of Radiology, Weill Cornell Medicine, New York, NY, United States

**Keywords:** self-assembly, biomolecules, drug delivery, nanoparticles, hybrid materials

Self-assembled materials generated by different non-covalent interactions have demonstrated tremendous advantages in healthcare applications due to their ease of preparation, potential for multifunctionality, and good biocompatibility. The definition of biomolecules covers macromolecules, small molecules, and ions that are naturally present in biological system or essential for the life of living organisms. Typical biomolecules include the proteins/peptides, nucleic acids, lipids and carbohydrates, which could efficiently mediate profound noncovalent interactions including hydrophobic interaction, electrostatic interaction, ion coordination, hydrogen bonding, etc. Based on these multiple noncovalent interactions, the hierarchical assembly of biomolecules contributes to the complex structure of cell, while it can also be engineered to fabricate biomaterials of different scales. One typical example is nanomaterials based on the self-assembly of biomolecules, which has been widely explored as therapeutics, biomaterials, sensors, imaging agents and food supplements, etc. The assembly of synthetic molecules and their hybrid assembly with biomolecules can further expand the biomedical applications. This topic mainly discusses the self-assembled biomolecules and their healthcare applications. Three research articles and 3 reviews cover the self-assembly of lipids, peptides, synthetic polymer and the hybrid assembly of both organic and inorganic materials for drug delivery, imaging and beyond. When compared with the free drugs or building blocks for the assembly, these self-assembled nanomaterials greatly facilitate the therapeutic or diagnostic outcome under the different healthcare application settings.

The liposome that mainly assembled based on the hydrophobic interaction of amphiphilic lipids represents the most successful self-assembled nanomaterials for clinical translation since several liposome-based formulations have been used in clinics. Wu et al. successfully explored the liposome containing N-oleoylethanolamine (OEA), a naturally existed lipid acting as an agonist for the endogenous peroxisome proliferator activated receptor alpha (PPAR-α), to improve the therapeutic index for stroke. While the poor solubility of OEA significantly limited its bioavailability and therapeutic efficacy, sustained release of OEA could be realized through incorporating it within the liposome. Compared with OEA only, this liposomal formulation of OEA demonstrated multiple benefits including protecting neurons from apoptosis, alleviating inflammation and improving the survival rate of focal cerebral ischemia rat. Considering all the components are biocompatible, the OEA loaded in liposome provides a promising strategy for anti-stroke therapy. Host-guest interaction is another non-covalent interaction that have been intensively researched to construct supramolecular materials of various functions. Xiao et al. utilized the host-guest interaction between Cucurbit [8] uril and PEG-APTS to design a supramolecular fluorescent materials, Q [8]@PEG-APTS, for monitoring acute kidney injury (AKI) through fluorescence imaging *in vitro*. Although this strategy provides a facile and biocompatible imaging agent to potentially diagnose AKI, the *in vivo* performance requires further verification. Polylactic acid (PLA) is a highly biodegradable polymer. In the review by Chen et al., various preparation strategies for assembling PLA with distinctive surface topographies are discussed in detail. The review highlights these assembled PLA bio-interfaces as a versatile platform for biomedical applications, including controlled drug release, targeted delivery, tissue scaffold and phototherapy. As many of these biomedical applications potentially relied on PLA and its self-assembly processes, the preparation strategies are summarized and perspectives are provided.

While the above contributed articles are focusing on the biomedical applications of the self-assembled materials, an efficient and intelligent self-assembly could potentially afford improved outcome for healthcare purposes. Basically, rational design of the assembly of biomolecules, synthetic component and the combination of both organic and inorganic materials are highly desirable. Dias et al. reported a hierarchically self-assembled structure based on reflectin-derived peptide. The reflectins are disordered proteins that are naturally linked to the cephalopod camouflage, thus their derived materials potentially demonstrate excellent optic and electronic properties. By utilizing the reflectin-derived protopeptide YMDMSGYQ as a building block, nanofibers and hydrogels could be generated through hydrophobic interactions, π–π stacking and hydrogen bonding, etc. This hierarchical assembly not only provides insights into the reflectin assembly mechanisms but also new options to prepare the reflectin-based materials. Similarly, Fang et al. reviewed the preparation strategies of macrocyclic complexes through the metal ion coordination of foldable and amphiphilic ligands. This type of self-assembled materials can be engineered to produce predictable and controllable structures and are emerging for biosensing and therapeutic applications. While the preparation of metal-coordination-based macrocyclic complexes is facile and cost effective, future challenges and research direction have also been discussed regarding to the biomedical applications. The self-assembly strategies could also be adopted to build hybrid materials that composed of both organic and inorganic materials. Song et al. contributed a review of “Hierarchy of hybrid materials. Part-II: The place of organics-on-inorganics in it, their composition and applications” with a focus on functionalization of inorganic materials with organic molecules as well as their applications. The organic materials involved in the hybrid materials are mainly the biomolecules (proteins, nucleic acids, carbohydrates, etc.) and other organic additives, while the inorganic parts can be metal, metal oxide, metal-organic framework and carbon nanoparticles. Unlike the biomolecules-based materials only, the incorporation of inorganic materials adds complement properties into the hybrid materials and thus has been widely used in biomedicine and beyond.

To summarize, this topic covers the frontiers of self-assembled materials based on biomolecules as well as its combination with synthetic materials. With the rapid development of materials in chemistry, biology and medicine, the healthcare applications of self-assembled biomolecules are far beyond the articles discussed in this topic. Nevertheless, the ultimate goal of the self-assembled biomaterials should be their translation which could be realized through the rational design of the assembly as well as a thorough evaluation of efficacy and safety for different biomedical applications.

